# The Role of Technological Rehabilitation in Patients with Intensive Care Unit Weakness: A Randomized Controlled Pilot Study

**DOI:** 10.3390/jcm12072612

**Published:** 2023-03-30

**Authors:** Letizia Castelli, Chiara Iacovelli, Augusto Fusco, Vincenza Amoruso, Cristina Cuccagna, Claudia Loreti, Silvia Giovannini, Luca Padua

**Affiliations:** 1 UOC Neuroriabilitazione ad Alta Intensità, Fondazione Policlinico Universitario A. Gemelli IRCCS, 00168 Rome, Italy; 2 Department of Emergency, Anaesthesiology and Intensive Care Medicine, Fondazione Policlinico Universitario A. Gemelli IRCCS, 00168 Rome, Italy; 3 Department of Geriatrics and Orthopaedics, Università Cattolica del Sacro Cuore, 00168 Rome, Italy; 4 UOS Riabilitazione Post-Acuzie, Fondazione Policlinico Universitario A. Gemelli IRCCS, 00168 Rome, Italy

**Keywords:** rehabilitation, robotics, virtual reality, vibration, brain injury, intensive care units

## Abstract

Intensive-Care-Unit-Acquired Weakness (ICU-AW) is the most common neuromuscular impairment in critically ill patients and can have a significant impact on long-term disability. Early rehabilitation has been suggested to facilitate the natural recovery process. This is a pilot, randomized, single-blind study that aimed to evaluate the effectiveness of intensive combined technological rehabilitation treatment including focal muscle vibration and non-immersive virtual reality for patients with severe acquired brain injury (sABI) and ICU-AW. Twenty-four patients were randomized into the conventional group, which performed only conventional rehabilitation, and the experimental group, which also performed technological treatment. At baseline and after 3 weeks of treatment, assessments of motor function, autonomy, disability and quality of life were conducted. At the end of the intervention, both groups showed significant improvements. However, patients in the experimental group achieved greater improvements in disability (*p* = 0.001) and quality of life (*p* = 0.001). The results show that intensive structured rehabilitation is effective in improving the motor function, disability and quality of life of patients with severe acquired brain injury and acquired weakness. The combination of non-immersive virtual reality training and focal muscle vibration can result in a significant improvement in overall disability and quality of life compared with conventional treatment alone.

## 1. Introduction

Intensive-Care-Unit-Acquired Weakness (ICU-AW) is the most common neuromuscular impairment in critically ill patients. Critical Illness Polyneuropathy (CIP), Critical Illness Myopathy (CIM) and muscle atrophy contribute in varying proportions to ICU-AW [1].

There is a great scientific interest in ICU-AW, especially to identify possible risk factors. The pathophysiology of ICU-AW requires invasive procedures and carries potential risks for patients. Based on evidence in animal models, it can be inferred that ICU-AW is related to endogenous and biological factors and to alterations in the central nervous system, the peripheral nerves and the myofibers, and to the effects of the use of neuromuscular blocking agents [2,3,4]. A model for predicting the risk of ICU-AW in adults hospitalized in intensive care units (ICUs) has recently been developed, which includes five risk factors: gender, shock, time of mechanical ventilation, length of stay in the ICU and age [5].

ICU-AW conditions usually present with flaccid, symmetrical paralysis/paresis, especially in the lower limbs, with muscle weakness, often associated with visible atrophy [6]. Additionally, respiratory muscles are affected, resulting in difficulty in weaning from the ventilator [7,8].

ICU-AW has a significant impact on the long-term disability of patients with severe acquired brain injury (sABI) [9]. Muscle atrophy and reduced muscle strength are significantly greatly exacerbated by immobility during critical illness. As a result, patients may lose half of their muscle mass, resulting in severe physical impairment [10]. Some authors have suggested how early mobilization can be an effective method to accelerate the natural recovery process in ICU-AW [11] and the important role of caregivers [12]. More recently, a meta-analysis established that early mobilization is effective in increasing strength and functional status [13]. In addition, Watanabe and colleagues demonstrated the effectiveness of a long-term protocol involving daily 20 min cycle ergometer training in addition to conventional treatment [14]. A recent study (the ExPrES study) evaluated that the combination of neuromuscular electrical stimulation, high-protein supplementation, mobility and rehabilitation attenuates lower limb muscle loss in ICU patients [15].

ICU-AW significantly affects the long-term disability of patients with sABI [9]. The term sABI describes a wide range of neurological diseases that occur after birth. Patients may recover differently from brain injuries depending on their type and location, and the extent of the injuries [16]. Impairments can range from mild, reversible symptoms to significant impairments with prolonged disorders of consciousness, causing a wide range of disabilities [17]. The nature of these disabilities may also change over time [18]. Various tailored interventions are used in the rehabilitation treatment of patients with sABI to restore cognitive and motor functions. The increasing use of new technologies allows for the incorporation of meaningful, repetitive, intensive and task-specific training in an enriched environment, considered a key factor in neurological rehabilitation. This involves the use of robotics [19,20,21,22], virtual reality [20,23] and muscle vibration [24,25,26].

The use of robotics and virtual reality integrated with muscle vibration can be considered an acceptable complementary tool for comprehensive and multifaceted rehabilitation [27]. Immersive reality-based treatments can also improve motor outcomes in the rehabilitation of patients with sABI by promoting physiological activation of brain areas related to motor learning [28]. Enriched virtual reality (VR) training can be used to provide repetitive practice with multisensory stimuli (audio, visual, motor, proprioceptive), maximizing neuroplasticity processes, motor learning and the overall recovery of sensorimotor performance [29,30]. Specifically, focal muscle vibration (FMV) has been shown to be an effective neurorehabilitation tool, reducing spasticity and promoting motor learning within a functional activity, regardless of the etiology of the neurological disease [31]. It has been argued that FMV can enhance learning-dependent plasticity processes within sensory–motor areas. The integration of two different synaptic inputs at the same time can produce a kind of associative plasticity [28,32].

Nevertheless, the use of integrated technologies is still limited in individuals with ICU-AW. While several studies have shown that early rehabilitation interventions such as muscle strengthening were helpful, two systematic reviews with meta-analyses found no conclusive evidence of their effectiveness following discharge from the ICU [33]. The possible failure of this rehabilitation could be related to the too light interventions proposed (early mobilization, passive exercise, bedside cycle ergometer, chair sitting, electrical stimulation), and also to the context of intervention (intensive care).

The purpose of this study is to evaluate the effectiveness of an intense multidimensional rehabilitation treatment based on combined technological—FMV and non-immersive VR—and conventional (physical therapy) treatments, for patients with sABI and ICU-AW in an intensive neurorehabilitation unit.

## 2. Materials and Methods

### 2.1. Protocol

This is a randomized, single-blind pilot study (Registration number: NCT05464160 on clinicaltrials.gov). The present research was conducted from December 2021 to May 2022, and included patients with sABI outcomes admitted to the High-Intensity Neurorehabilitation Unit of the Fondazione Policlinico Universitario “A. Gemelli” IRCCS who met the inclusion criteria.

Inclusion criteria were: (i) age between 40 and 70 years [34]; (ii) sABI due to vascular or traumatic etiology; (iii) latency from the acute event between 20 days and 3 months; (iv) presence of ICU-AW, including CIM and CIP, detected by clinical evaluation, defined as generalized flaccid muscle weakness, exceeding that due to “central” motor involvement with Medical Research Council Sum Score (MRC Sum Score) < 48 [35], in absence of other causes of muscle weakness, such as preexisting neuromuscular disorders and/or rhabdomyolysis; and (v) electrophysiological diagnosis of ICU-AW, i.e., evidence of motor and sensory axonal polyneuropathy and/or myopathic motor unit potentials with or without fibrillation potentials on needle examination.

In contrast, patients with the following characteristics were excluded: (i) deep vein thrombosis; (ii) venous accesses on the limbs to be treated; (iii) oncological diseases; (iv) epilepsy; (v) open skin lesions and/or local infections and/or sepsis; (vi) previous stroke and/or neurodegenerative diseases (such as Parkinson’s, motor neuron disease, etc.).

Patients who met the criteria were included in the study and were randomized according to a computer-generated 1:1 allocation ratio Random Sorting procedure using PASS2019 software.

### 2.2. Interventions

Patients included in the study were randomized into two groups: the experimental group (Expe-G) and the conventional group (Conv-G). The Expe-G performed structured, technological treatment with Omego^®^ and FMV for 5 days a week, for a total of 3 weeks, in addition to the conventional treatment provided by their clinical condition. Patients in the Conv-G were treated with conventional rehabilitation interventions for the 3 weeks under study, according to the common treatments for ICU-AW patients (passive rehabilitation in bed, cycle ergometer, chair sitting) with the same duration as the Expe-G session.

Omego^®^ (Tyromotion, Graz, Austria) is a robotic device for lower limb therapy using non-immersive VR. It consists of a cycle ergometer suitable for lower limb training, which allows testing of muscle recruitment, range of motion, coordination and perception. Specific computer software calibrates the training program as desired. A large display on a flat screen shows a virtual scenario in a non-immersive mode.

FMV was applied through a non-invasive device, EVM EVO (Endomedica Srl, Rome, Italy), which exploits the effect of sound waves on tissues by rapidly alternating air pressures and depressions, with a frequency range of 30 to 300 Hz, in order to stimulate nerve and muscle structures.

The conceptual framework of the research was developed according to the frequency, intensity, time, type (FITT) principles of therapeutic modalities for physical rehabilitation [36,37]. The method was developed in terms of frequency of use, intensity, type of intervention and time in order to structure a logical treatment protocol. These variables can be manipulated according to the functional, physiological and psychological requirements of the research objective.

**Figure 1:**
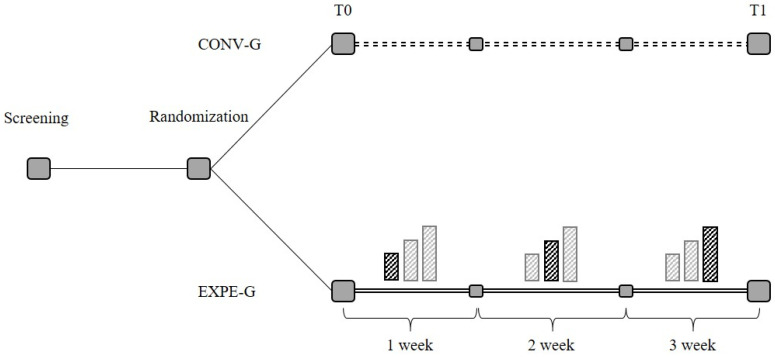
Design of the study.

The experimental treatment of Expe-G, lasting 5 days per week for a total of 3 weeks, was organized as follows: (i) first week: 10 min of treatment with EVM EVO, applied to the lower limbs with a frequency of 300 Hz plus 10 min of treatment with Omego^®^ in active-assisted mode, for 20 min of total treatment; (ii) second week: 15 min of treatment with EVM EVO, applied to the lower limbs with a frequency of 300 Hz plus 15 min of treatment with Omego^®^ in active-assisted mode, for a total of 30 min of treatment; (iii) third week: 20 min of treatment with EVM EVO, applied to the lower limbs with a frequency of 300 Hz plus 20 min of treatment with Omego^®^ in active-assisted mode, for a total of 40 min of treatment. Figure 1 shows the study design.

### 2.3. Assessment

Clinical assessments were performed at onset (baseline, *T*0) and after 3 weeks (*T*1). At baseline, the following information was collected: age, gender, schooling, comorbidities, latency from the acute event, classification of disorder of consciousness and current drug therapies. Both at baseline and at the end of treatment, the following rating scales were performed: (i) Motricity Index Lower Limb (MI-LL); (ii) Trunk Control Test (TCT); (iii) Functional Ambulation Classification (FAC); (iv) modified Barthel Index (mBI); (v) EuroQol-5 dimension (EQ-5D); (vi) Disability Rating Scale (DRS).

MI-LL is a motor component assessment test that examines three lower extremity movements, specifically ankle dorsiflexion, knee extension and hip flexion. The score ranges from 1 to 100 [38].

TCT assesses trunk control. It consists of four items: ability to turn on the sick side, to turn on the healthy side, to move from supine to sitting position and to balance from sitting on the edge of the bed [39].

The FAC scale measures the walking ability of inpatients. The score ranges from 0 to 5, where 0 indicates non-functional ambulation and 5 indicates independent ambulation [40].

The mBI is a tool that assesses a patient’s functional independence. It assesses 10 items and the overall score ranges from 0 (total dependence) to 100 (total independence) [41].

The EQ-5D is a quality of life questionnaire that includes five dimensions: mobility, self-care, usual activities, pain/discomfort and anxiety/depression. Each dimension has five levels, from “no problems” to “extreme problems” [42].

The DRS is an instrument that assess a patient’s disability through four domains: (i) vigilance, awareness and responsiveness, (ii) cognitive ability for self-care activities, (iii) functional level and (iv) employability. The score ranges from 0 to 30, where 0 indicates no disability and 30 means death. Ten categories can be identified based on the DRS score: from “no disability” (category 1) to “death” (category 10) [43].

### 2.4. Statistical Analysis

Due to the exploratory nature of this pilot study, no sample size analysis was performed. Data are presented as mean (standard deviation) or median (range), as appropriate. Quantitative variables were summarized with mean and standard deviation (SD), median and interquartile range (IQR) where appropriate. Qualitative variables were presented in absolute and percentage frequency tables.

The Shapiro–Wilk probability test was used to assess the normality of the distributions. Within-group analysis was based on the application of the Wilcoxon Signed Rank test for each clinical outcome recorded at *T*0 and *T*1. Between-group differences were analysed by comparing the percentage increase in each outcome, defined as:

$$\Delta S=\frac{S\left(T1\right)-S\left(T0\right)}{S\left(T0\right)}$$

where *S* is one of the clinical or balance outcomes used in the study (except FAC), and *S*(*T*0) and *S*(*T*1) are the *S* scores at *T*0 and *T*1, respectively.

The between-group analysis of the FAC scale was conducted by considering the differences between the scores, [*S*(*T*1)—*S*(*T*0)], because the minimum value of these scales is 0 and normalization was therefore not possible. The Mann–Whitney U test was applied to compare the percentage increase calculated for each group. Statistical significance for each test was set at *p* = 0.05.

Statistical analysis was performed with SPSS (IBM SPSS Statistics for Windows, version 25.0, Armonk, NY, USA).

## 3. Results

**Figure 2:**
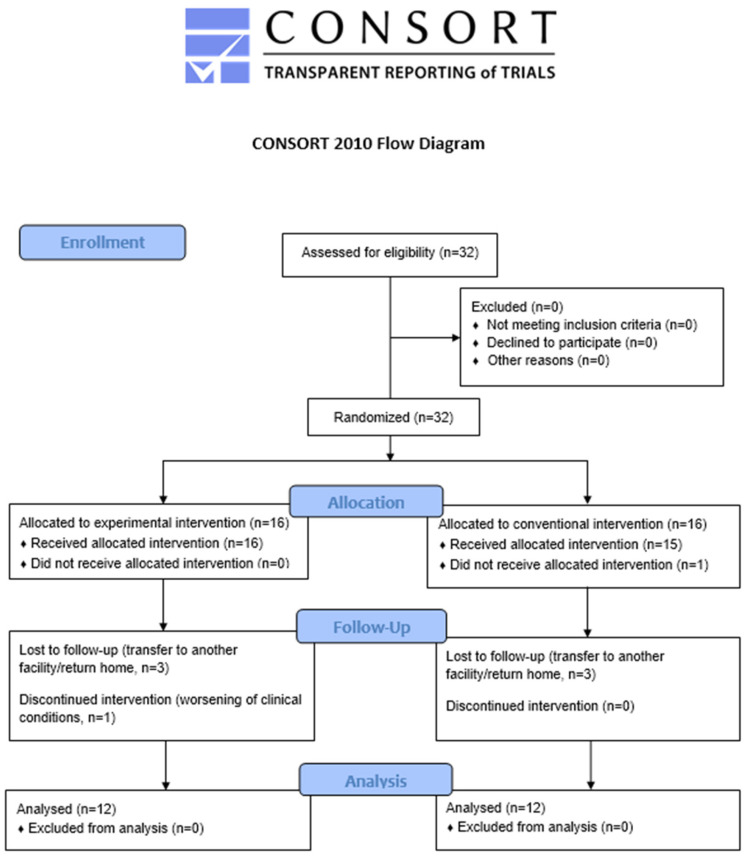
The CONSORT flowchart.

Twenty-four patients with sABI admitted to the High-Intensity Neurorehabilitation Unit, whose characteristics are shown in Table 1, were evaluated and included in the study from December 2021 to May 2022. To better describe the study results and the process performed, we decided to follow the consolidated standard of reporting trial (CONSORT) flowchart (Figure 2), as suggested by the Enhancing the QUAlity and Transparency Of health Research (equator network) [44]. The two treatment groups, Expe-G and Conv-G, were comparable in terms of demographic and clinical characteristics at baseline (*p* values > 0.2).

Intragroup analysis showed statistically significant improvements in all clinical scales in both groups between *T*0 and *T*1 (Table 2).

**Figure 3:**
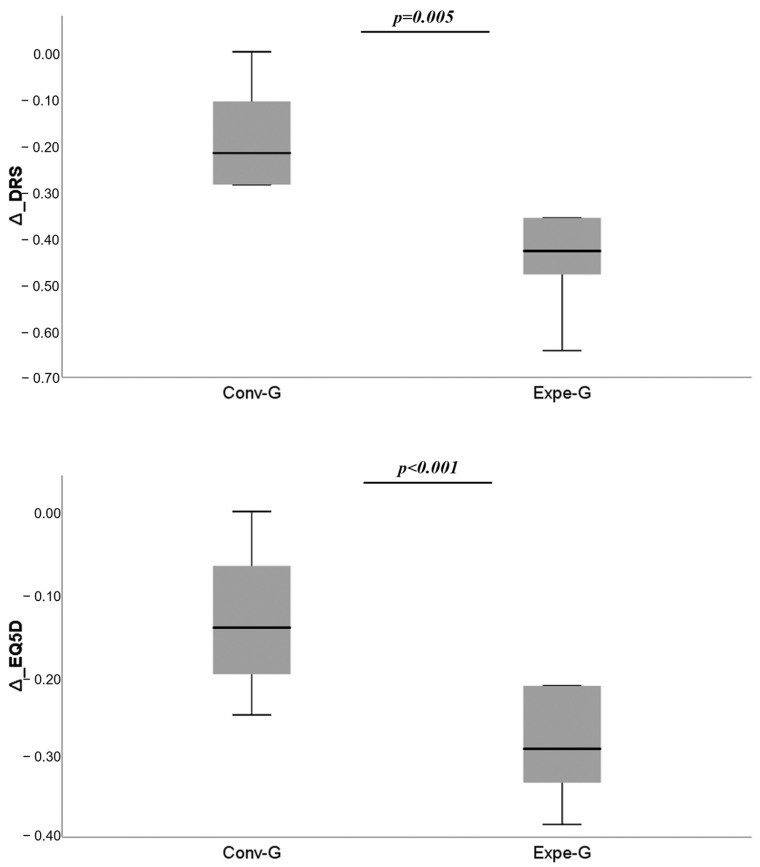
The comparisons of change (Δ) for DRS (disability) and EQ-5D (quality of life) show significant differences between Expe-G and Conv-G.

In the between-group comparison, a statistically significant difference was found only in DRS (*p* = 0.001) and EQ-5D (*p* < 0.001), where there was a greater improvement in the experimental group (Figure 3).

## 4. Discussion

In this pilot study, it was evaluated whether the implementation of conventional physical therapy with a robotic treatment with non-immersive VR combined with FMV of the lower limbs was more effective than conventional rehabilitation treatment alone in patients with sABI and ICU-AW. The underlying hypothesis was that this intensive-type rehabilitation approach could improve the recovery of lower limb function, reduce overall disability and improve the quality of life of hospitalized patients.

The emerging results partially support this hypothesis. This pilot study of 24 patients found significant improvement in all scales in both groups (both experimental and control), but patients in the experimental group showed a statistically significant improvement in disability, as assessed by the DRS (*p* = 0.001), and quality of life, as assessed by the EQ-5D (*p* < 0.001). The substantial improvement in all evaluated outcomes could be attributed to the intensive rehabilitation used in neurorehabilitation. Most of the studies on ICU-AW have been conducted in intensive care units. Early rehabilitation during the ICU stay may not have an “intensive” trait (a parameter measured by the frequency of sessions, the duration of interventions performed in each session and the intensity of these interventions), probably due to the lack of stability of patients’ clinical conditions. Therefore, the potential benefit of early rehabilitation, in the sense of early movement or functional exercises, could be hampered by cardiovascular, respiratory and neurological conditions [33,45]. The use of integrated rehabilitation, such as that typically provided in a neurorehabilitation setting, can successfully complement traditional methods of treating patients with sABI.

No significant differences were found between the experimental and conventional rehabilitation protocol in terms of functional improvement, understood as improvement in muscle strength, assessed by MI-LL, posture, assessed by TCT, and gait, assessed by FAC. In the experimental group, data analysis showed a significant improvement in terms of disability, which could be specifically determined by the use of combined conventional, technological and mechanical treatments (VR and robotic systems in addition to FMV). A combination of these interventions can provide optimal synergy to achieve complex motor outcomes [46], improving lower limb functional activity, balance, gait and overall fitness [47,48]. Individuals with severe brain injury may be impaired by persistence arousal disturbance. Rehabilitation aims to improve awareness and responsiveness to various stimuli, cognitive ability for self-care activities, autonomy in daily living and psychosocial adaptability. In a recent study, the authors documented how the use of VR can improve the recovery of cognitive function in patients with sABI while also improving muscle recruitment, articulation, coordination and perception [20].

Surprisingly, a similar statistical difference between the groups was not found in the assessment of autonomy using mBI. Possible reasons for this non-statistical difference between the groups include the type of outcome measures and the small sample size. In particular, the mBI is widely used by rehabilitation professionals to assess autonomy in different diseases and settings (acute care, rehabilitation and community) [30]. Compared with the DRS, the mBI focuses more on the level of autonomy in activities of daily living. Therefore, it is possible that the DRS, a specific scale designed for individuals with sABI, was more sensitive in recording differences between assessment moments in this type of patient.

FMV was also used in this pilot study. The combined rehabilitation approach of FMV and non-immersive VR has been successfully used in the treatment of upper limb spasticity and motor function in people with stroke outcomes [28]. It has been argued that the amount of information from muscle and joint receptors reaching the sensory and motor cortices during FMV may play a significant role in determining muscle reinforcement depending on the frequency of activation, also acting on primary sensory and motor areas [49]. Vibratory stimulus could also affect antagonist muscle activation by reducing its activity [50] due to an increase in mechanoreceptor activity generated by vibration [51]. In addition, the increase in tissue temperature could determine the efficiency of the joint system [52].

Another relevant finding from this study is the significant improvement in quality of life, as assessed by the EQ-5D, in the experimental group compared with the conventional group. Recent work reported that ICU-AW still impaired quality of life 10 years after hospitalization [53]. Accurate diagnosis is critical because ICU-AW could hinder the rehabilitation pathway during and after treatment, affecting overall outcomes [54,55]. There is wide heterogeneity in the diagnosis of ICU-AW, probably due to the inconsistent definitions of ICU-AW, the difficulty of detection and the usual activity outside of the ICU in heterogeneous settings [53,56]. The results of this pilot study may suggest how an early diagnosis of ICU-AW and a structured, technology-based rehabilitation program can have an immediate impact on self-perceived quality of life.

Integrating cognitive and motor elements during robotic training can improve motivation and concentration while also enabling a higher level of active patient participation during sessions [20,57].

Some limitations must be taken into account when evaluating this study. Since this is a pilot study, the results should be viewed with caution, as since it is preliminary data, further feedback is needed to confirm the starting hypothesis. The main limitation of the study is the small sample size. However, according to the Julious Practice Rules for pilot clinical trials [58], the inclusion of 12 subjects per group was estimated, for a total population of 24 subjects. In addition, the guidelines for pilot studies indicate that phase 2 clinical rehabilitation pilot studies on physical and cognitive interventions can begin with a convenience sample of at least six participants [59]. Another limitation of the study is the lack of follow-up, both at discharge and thereafter. Longitudinal studies suggest continued functional improvement even after discharge from rehabilitation [18].

Given the sample size, moreover, any comorbidities and neuroanatomical impairments were not assessed through second-tier imaging investigations or other prognostic factors, such as neurophysiological and laboratory tests, in accordance with the purpose of the study. With a larger sample size, wider factors could be considered.

## 5. Conclusions

The results of our study indicate that an intensive rehabilitation course (both conventional and experimental) is effective in bringing about significant improvements in the lower limb muscle strength, posture, gait, disability and quality of life of patients with sABI and ICU-AW. Compared with the conventional therapy alone, the combination of conventional physical therapy, robotic lower limb training and non-immersive VR in addition to FMV results in significant improvements in overall disability and quality of life. These data reinforce the value of intensive rehabilitation in producing clinical effects in terms of motor function and independence in activities of daily living, also influencing the quality of life. The use of multitarget treatments can help overcome the different impairments associated with ICU-AW. The use of robotic and virtual reality systems can be easily exploited in different settings, accelerating access to comprehensive rehabilitation. However, further randomized trials are required to verify the effectiveness of this rehabilitation training in a larger population sample with longer follow-up.

## Figures and Tables

**Table 1:** Characteristics of the population at baseline.

		Expe-G (n = 12)	Conv-G (n = 12)	*p*-Value
Gender, n	Female vs. Male	4 vs. 8	7 vs. 5	0.321
Age, years	mean ± SD	63.75 ± 12.29	62.75 ± 8.29	0.749
Latency from acute event, days	mean ± SD	80.58 ± 13.77	70.25 ± 19.51	0.421
DRS	mean ± SD	14.83 ± 1.53	15.58 ± 4.80	0.279
MI-LL dx	mean ± SD	43.67 ± 7.87	45.83 ± 26.63	0.472
MI-LL sx	mean ± SD	42.00 ± 5.83	41.67 ± 15.04	0.329
TCT	mean ± SD	29.17 ± 17.65	28.17 ± 20.33	0.588
EQ-5D	mean ± SD	12.00 ± 2.13	12.67 ± 2.87	0.329
FAC	mean ± SD	0.25 ± 0.45	0.17 ± 0.39	0.755
mBI	mean ± SD	16.92 ± 10.18	19.17 ± 10.94	0.559

Expe-G, experimental group; Conv-G, conventional group; DRS, Disability Rating Scale; MI-LL, Motricity Index Lower Limb; TCT, Trunk Control Test; EQ-5D, EuroQoL-5 dimension; FAC, Functional Ambulation Classification; mBI, modified Barthel Index.

**Table 2:** The table shows the clinical scale’s values (mean and standard deviation) at *T*0 and *T*1, and its intragroup statistical analysis results (*T*0 vs. *T*1) for both Expe-G and Conv-G.

	Expe-G			Conv-G		
	*T*0 (Mean ± SD)	*T*1 (Mean ± SD)	*p*-Value (*T*0 vs. *T*1)	*T*0 (Mean ± SD)	*T*1 (Mean ± SD)	*p*-Value (*T*0 vs. *T*1)
**MI-LL dx**	43.67 ± 7.87	58.83 ± 13.03	**0.002**	45.83 ± 26.63	57.33 ± 25.49	**0.007**
**MI-LL sx**	42.00 ± 5.83	50.33 ± 16.02	**0.011**	41.67 ± 15.04	49.08 ± 18.72	**0.007**
**TCT**	29.17 ± 17.65	81.50 ± 14.86	**0.002**	28.17 ± 20.33	54.33 ± 25.27	**0.007**
**EQ-5D**	12.00 ± 2.13	8 ± 11.92	**0.002**	12.67 ± 2.87	11.17 ± 3.30	**0.002**
**FAC**	0.25 ± 0.45	2.42 ± 1.24	**0.002**	0.17 ± 0.39	1.75 ± 1.60	**0.003**
**DRS**	14.83 ± 1.53	8.33 ± 1.37	**0.002**	15.58 ± 4.80	12.50 ± 5.57	**0.003**
**mBI**	16.92 ± 10.18	41.75 ± 34.13	**0.002**	19.17 ± 10.94	34.58 ± 28.70	**0.007**

Expe-G, experimental group; Conv-G, conventional group; DRS, Disability Rating Scale; MI-LL, Motricity Index Lower Limb; TCT, Trunk Control Test; EQ-5D, EuroQoL-5 dimension; FAC, Functional Ambulation Classification; mBI, modified Barthel Index. In bold the *p*-values greater than 0.05.

## Data Availability

Data supporting the results are not available.
